# Cell Reprogramming to Model Huntington’s Disease: A Comprehensive Review

**DOI:** 10.3390/cells10071565

**Published:** 2021-06-22

**Authors:** Ruth Monk, Bronwen Connor

**Affiliations:** Centre for Brain Research, Department of Pharmacology and Clinical Pharmacology, Faculty of Medical and Health Sciences, School of Medical Science, University of Auckland, Auckland 1023, New Zealand; r.monk@auckland.ac.nz

**Keywords:** cell reprogramming, pluripotent stem cells, direct cell reprogramming, disease modelling, Huntington’s disease, striatal differentiation

## Abstract

Huntington’s disease (HD) is a neurodegenerative disorder characterized by the progressive decline of motor, cognitive, and psychiatric functions. HD results from an autosomal dominant mutation that causes a trinucleotide CAG repeat expansion and the production of mutant Huntingtin protein (mHTT). This results in the initial selective and progressive loss of medium spiny neurons (MSNs) in the striatum before progressing to involve the whole brain. There are currently no effective treatments to prevent or delay the progression of HD as knowledge into the mechanisms driving the selective degeneration of MSNs has been hindered by a lack of access to live neurons from individuals with HD. The invention of cell reprogramming provides a revolutionary technique for the study, and potential treatment, of neurological conditions. Cell reprogramming technologies allow for the generation of live disease-affected neurons from patients with neurological conditions, becoming a primary technique for modelling these conditions in vitro. The ability to generate HD-affected neurons has widespread applications for investigating the pathogenesis of HD, the identification of new therapeutic targets, and for high-throughput drug screening. Cell reprogramming also offers a potential autologous source of cells for HD cell replacement therapy. This review provides a comprehensive analysis of the use of cell reprogramming to model HD and a discussion on recent advancements in cell reprogramming technologies that will benefit the HD field.

## 1. Introduction

Huntington’s disease (HD) is a devastating neurodegenerative disorder for which there is currently no effective treatment. Despite the identification of the genetic cause of HD in 1993, the specific mechanisms driving the preferential degeneration of striatal medium spiny neurons (MSNs) remain elusive [1]. The high clinical failure rate of therapeutic interventions for HD may be attributable to the inadequacies of traditional HD models to replicate HD neuropathogenesis as it occurs in humans. The establishment of a well-characterized live human model of HD in a clinically relevant cell type may be the key to uncovering the undoubtedly complex molecular and cellular mechanisms driving HD.

HD human post-mortem brain tissue and animal and cellular models of HD have contributed enormously to the current understanding of HD neuropathogenesis. However, the study of HD in these systems has been unable to generate an effective treatment to slow or prevent disease progression. While being the gold-standard tool to assess HD neuropathology, HD human post-mortem brain tissue provides little insight into the mechanistic progression of HD. Although animal models address this by allowing for the study of disease pathology in a live system, no animal model can recapitulate the features of HD that are unique to humans, thus limiting clinical translatability.

In 1998 came the ground-breaking discovery that pluripotent stem cells (PSCs) could be isolated from the inner cell mass of donated human blastocytes and propagated almost indefinitely in vitro [2]. For the first time, scientists had a theoretically inexhaustible source of human embryonic stem cells (hESCs) capable of giving rise to live cell types that are typically inaccessible in living donors. While hESCs are considered the gold standard of pluripotency, their embryonic origin is controversial. In 2007 came the Nobel prize-winning discovery that human somatic cells from living donors could be reprogrammed to an hESC-like state via retroviral transduction of *OCT4*, *SOX2*, *KLF4*, and *cMYC* [3]. The generation of induced pluripotent stem cells (iPSCs) rapidly advanced the ability to study human development and pathology in a cell-type-specific and personalised manner.

## 2. Pluripotent Stem Cell Models of Huntington’s Disease

### 2.1. Human Embryonic Stem Cell Models of Huntington’s Disease

The revolutionary ability of cell reprogramming to generate human neurons in vitro for disease modelling was catalysed by the discovery that hESCs could be isolated from embryos affected by genetic disorders [2,4]. Since 2005, dozens of HD hESC lines have been generated from HD-affected embryos following pre-implantation genetic diagnosis, mostly with CAG lengths within the range of adult-onset HD (37–51 CAG) [4,5,6,7,8,9,10,11,12,13,14,15]. ‘HD’ hESCs have also been generated using genetic modification of normal hESCs to produce a series of isogenic HD hESC lines with 45–74 CAG repeats [16] and even up to 145 CAG repeats [17]. Importantly, HD hESCs can be differentiated into neurons and used to investigate HD-associated phenotypes (Table 1). HD hESCs differentiate into neurons with no difference in neuronal yield [6,18,19] or neurite length [19] compared to normal hESCs. However, McQuade et al. reported significantly higher yields of GABAergic neurons and increased neurite branching in HD compared to normal hESC-derived neurons [19]. Niclis et al. also reported increased intracellular calcium and glutamate-evoked responses in HD hESC-derived neurons (51 CAG), although the expression of HD-associated genes was similar in HD and normal hESCs throughout the time course [18].

Although considered a hallmark feature of HD, mHTT aggregates were only reported in genetically modified HD hESC-derived neurons [16,17]. This may be due to the lower CAG repeat length of most HD hESC lines compared to the genetically modified counterparts that possess juvenile CAG repeat lengths. Furthermore, cell death was only observed in HD or genetically modified HD hESC-derived neurons upon kinase inhibition [19] or growth factor withdrawal [17]. This suggests that HD hESC-derived neurons may be limited in their capacity to recapitulate a degenerative phenotype. Interestingly, disease-associated phenotypes have been reported in hESCs and hESC-derived neural progenitor/precursor cells (NPC) in HD and genetically modified HD cell lines. While mitochondrial dysfunction was not observed in HD hESC-derived neurons, it was present in HD hESCs [19] and HD hESC-derived NPCs [20]. Genetically modified HD hESCs also displayed dysregulated mitotic spindle orientation and impaired neural rosette formation [16], although neural rosette formation was normal in innate HD hESCs [20]. 

There are mixed reports regarding the stability of the CAG repeat length upon hESC differentiation. Niclis et al. reported CAG repeat length instability upon neuronal differentiation [6], yet Seriola et al. observed CAG repeat length stability upon differentiation to all three primary germ layers [21]. Ruzo et al. also reported aneuploidy in genetically modified HD hESCs, the severity of which correlated with the length of the CAG repeat [16]. Interestingly, Seriola et al. observed the downregulation of DNA mismatch repair genes upon neural induction of both normal and HD hESCs [21]. Together, this indicates that the differentiation process itself may interfere with the phenotype of hESC-derived cells, with the CAG repeat expansion exacerbating these effects. 

### 2.2. Induced Pluripotent Stem Cell Models of Huntington’s Disease

The advent of cell reprogramming revolutionised the field of disease modelling. For the first time, neurons from individuals with HD could be studied *in vitro*, without the ethical concerns or limited availability of HD hESCs. To date, HD iPSC cell lines have been generated from individuals with 40–180 CAG repeats, predominantly via viral delivery of *OCT4, SOX2, KLF4,* and *cMYC* or *OCT4, SOX2, KLF4, LMYC, LIN28,* and *NANOG,* or via episomal delivery of *OCT4, SOX2, KLF4, LMYC, LIN28*, and p53 shRNA (Table 2). Despite only accounting for 5% of all HD cases, juvenile HD with symptom onset by 20 years of age has been largely overrepresented in cell reprogramming models of HD. As the age of symptom onset is not always known for reprogrammed HD lines, those with at least 60 CAG repeats are widely regarded as potential juvenile HD [23]. Although there are clinical and neuropathological similarities between juvenile and adult-onset HD, including the preferential degeneration of MSNs, it is likely that there are some unique differences in the neuropathogenic mechanisms that contribute to neurodegeneration in juvenile and adult-onset HD [23]. While the nature and importance of these neuropathogenic differences are not yet well understood, the overrepresentation of juvenile HD reprogramming models presents a challenge when extrapolating the findings from juvenile HD lines to adult-onset HD, which makes up the vast majority of all cases with HD [23].

#### 2.2.1. Phenotypes Observed in Huntington’s Disease induced Pluripotent Stem Cells

Although HD iPSCs demonstrated similar proliferation rates [54] and numbers of mitotic cells [24] to normal iPSCs, increased chromosomal abnormalities [38] and altered expression of embryonic development genes [51] were observed in HD iPSCs (Table 3). There are discrepancies in the expression of DNA damage response genes in HD iPSCs, with some studies reporting decreased gene expression [49,51], and others reporting that DNA damage response gene expression was normalised in reprogrammed HD iPSCs [29,38]. The latter observation may be due to methylation changes that occur during reprogramming [29], particularly when reprogrammed using shRNA against p53 [38,49]. Nonetheless, Mollica et al. observed CAG repeat length stability in HD cell lines with adult-onset CAG repeat lengths when reprogrammed from fibroblasts [29].

Similar to HD hESCs, disease-associated phenotypes have been reported in HD iPSCs. The phosphorylation of ERK was reduced in HD iPSCs [50] in response to fibroblast growth factor (FGF) [53]. Compared to normal iPSCs, HD iPSCs demonstrated increased proteasome [32] and lysosomal activity [24]. Mitochondrial pathologies were also present in HD iPSCs, including reduced mitochondrial number, reduced mitochondrial area, and reduced OPA1 co-localisation, indicative of diminished mitochondrial fusion in HD [61]. HD iPSCs also exhibited metabolic and bioenergetic imbalances compared to control iPSCs [61]. Furthermore, increased expression of oxidative stress and antioxidant response genes and proteins [50,55] have been reported in HD iPSCs, as well as the reduced expression of oxidative stress response proteins [55]. While no difference in caspase-3/7 activity was observed with [54] or without [24] growth factor withdrawal, HD iPSCs displayed increased expression of caspase signalling genes [54]. HD iPSCs also demonstrated dysregulated expression of factors regulating p53 and apoptosis [51,55], with increased expression of p53, phosphorylated p53, and phosphorylated ATM [49]. These results are consistent with HD iPSCs facing considerable cellular stress, even in the iPSC stage. However, the inclusion of shRNA against p53 during reprogramming abolished this response, indicating that the reprogramming method itself influences the presence of disease-associated phenotypes in iPSCs [38,49].

#### 2.2.2. Phenotypes Observed in Huntington’s Disease Neural Precursor Cells Derived from Induced Pluripotent Stem Cells

Upon neural induction, HD iPSCs exhibit phenotypes consistent with altered early neurodevelopment, including reduced sphere formation [45] and abnormalities in neural rosette formation [34,47,68] (Table 4). These phenotypes were most frequently reported in juvenile HD lines, often correlating with the length of the CAG repeat [34,45,47,68]. Conforti et al. reported that HD iPSCs with 180 CAG repeats had the complete inability to acquire a neuroectodermal phenotype, most likely due to lack of intrinsic *OCT4* downregulation upon neural induction [47]. Furthermore, CAG repeat length expansion has been reported in one juvenile HD iPSC line upon neural induction, increasing from 110 to 118 CAG repeats [45]. In contrast, CAG repeat lengths in HD iPSCs from adult-onset HD remained stable upon reprogramming and neural induction [29]. The expression of embryonic development, cell signalling, and cell assembly genes and proteins were altered in both adult-onset and juvenile HD iPSC-derived NPCs [45,54,59]. However, the altered expression of axon guidance genes was only reported in juvenile HD iPSC-derived NPCs [45,59]. Furthermore, juvenile HD iPSC-derived NPCs demonstrated altered expression of ventral and dorsal forebrain development genes, with most downregulated in HD [59]. Notably, the majority of neurodevelopmental phenotypes were observed in juvenile HD iPSC-derived NPCs, suggesting a prominent neurodevelopmental component underlying juvenile HD that may not be as relevant to the most common adult-onset form of HD.

In support of the reduced expression of *brain-derived neurotrophic factor (BDNF)* observed in HD post-mortem brain tissue and animal models [71,72,73,74,75], both An et al. [54] and Charbord et al. [57] reported reduced *BDNF* expression in juvenile HD iPSC-derived NPCs. In the latter study, the expression of *BDNF* was restored by RE1-silencing transcription factor (REST) inhibition, providing support for the role of altered REST expression in *BDNF* transcription in HD [57]. The reduced expression of transforming growth factor β (TGFβ) signalling genes and protein has also been reported in HD iPSC-derived NPCs [54,59,60], albeit inconsistently between studies and HD iPSCs [54]. Interestingly, alterations in calcium signalling genes were only observed in HD iPSC-derived NPCs from an HD line with 60 CAG repeats [45]. These results indicate that disease-associated phenotypes in HD iPSC-derived NPCs may depend on the CAG repeat length and the corresponding disease severity. Some of the phenotypes present in HD iPSCs persisted in HD iPSC-derived NPCs, including increased lysosomal activity [24], reduced proteasome activity [32], increased resistance to manganese cytotoxicity [40], metabolic alterations [61], and mitochondria number, density, and OPA1 co-localisation impairments [61]. Thus, it is unclear whether the presence of these phenotypes in HD iPSC-derived NPCs represents disease-associated changes during neurodevelopment or whether they reflect phenotypes that arose as a result of iPSC reprogramming itself. Interestingly, Lopes et al. observed that, while HD iPSCs had higher intracellular calcium levels compared to control iPSCs, the HD NPCs has lower levels compared to control NPCs, suggesting that the maintenance or alteration of different phenotypes within related cell function pathways between iPSC and NPCs is inconsistent [61].

Similar to HD iPSCs, most studies conducted in HD iPSC-derived NPCs did not report a degenerative phenotype, and increased caspase activity under standard culture conditions was only observed in HD iPSC-derived NPCs from a juvenile 180 CAG repeat line [45]. Upon growth factor withdrawal, HD iPSC-derived NPCs frequently demonstrated increased caspase-3/7 activity [53,54,59] and cell death [54]. These phenotypes were reversed by exogenous TGFβ administration [59,60] or in isogenic control lines [54]. Similarly, artificially-induced DNA damage increased the expression of apoptotic signalling factors, an effect reversed by ATM inhibition [49]. HD iPSC-derived NPCs also exhibited increased susceptibility to induced oxidative stress, although degenerative phenotypes were not observed under standard culture conditions [45]. These results indicate that the majority of HD NPC-specific phenotypes observed under standard conditions were only observed in juvenile HD lines, especially with very-long CAG expansions (>100 repeats), further highlighting the potential neuropathogenic differences that contribute to MSN degenerative and disease progression between juvenile and adult-onset HD.

#### 2.2.3. Neurodevelopmental and Neuronal Maturation Phenotypes Observed in Huntington’s Disease Neurons Derived from Induced Pluripotent Stem Cells

Following neural induction, HD iPSC-derived NPCs were differentiated into predominantly neurons (Table 5) or astrocytes [76,77,78]. iPSC-derived astrocytes exhibited large cytoplasmic vacuoles, possibly due to compromised autophagy [76], as well as electrophysiological impairments [78]. As MSNs undergo preferential degeneration in HD, the majority of HD iPSC studies used striatal differentiation protocols, albeit with varying success of DARPP32+ neuron generation (0–80% of neurons DARPP32+ [22,24,25,30,32,33,35,36,40,41,42,43,45,47,62,64,65,68]). Disease-associated phenotypes have also been investigated in iPSC-derived mixed [37,44,55,58] and cortical [40,63] neuronal cultures. 

Similar to HD iPSCs [51] and HD iPSC-derived NPCs [45,59], the expression of genes and proteins involved in cell growth, proliferation, function, signalling, and embryonic development is reportedly altered in HD iPSC-derived neuronal cultures [22,33,43,45,55]. Although neuronal yield is typically similar between normal and HD iPSC-derived neuronal cultures, there have been reports of reduced neuronal yield in juvenile HD [55], correlating with the length of the CAG repeat [47]. This is not consistent between studies, with Mattis et al. reporting increased neuronal yields in HD iPSC-derived neuronal cultures compared to normal [41]. Interestingly, Cohen-Carmon et al. only observed significant differences in the expression of genes involved in neurogenesis and the increased expression of GAD in juvenile HD iPSC-derived neuronal cultures when cells were exposed to progerin, an inducer of artificial aging [22].

Research conducted in juvenile HD iPSC-derived neuronal cultures has reported the reduced expression of general [63] and striatal [43,47] neurodevelopment and maturation genes. Reduced neurite length has also been observed in juvenile HD iPSC-derived neurons [55], both of cortical [63] and striatal [64] identities, with the reduction in neurite length correlated with the length of the CAG repeat in cortical neurons [63]. Juvenile HD iPSC-derived neuronal cultures contained higher yields of non-proliferative Nestin+ NPCs throughout differentiation, again indicative of abnormal neurodevelopment [41,44]. Consistent with this hypothesis, neurodevelopmental phenotypes in juvenile HD iPSC-derived neurons were reversed by treatment with the neurogenesis enhancer isoxazole-9 [43] or the Notch signalling inhibitor DAPT [44]. Similarly, Smith-Geater et al. demonstrated that adult-onset HD iPSC-derived neuronal cultures also contain a persistent population of NPC-like cyclin D1+ cells [33]. This population of cyclin D1+ cells in HD were abrogated by Wnt inhibition during differentiation [33]. Interestingly, the expression of striatal development genes was increased in HD iPSC-derived neuronal cultures from individuals with adult-onset CAG repeat lengths [30], consistent with the increased proportion of GABAergic neurons in hESC-derived neuronal cultures from embryos with adult-onset HD CAG repeat lengths [19]. Together, this may indicate that both juvenile and adult-onset HD iPSC-derived neurons exhibit altered neurodevelopment, with the differential nature of these phenotypes influenced by CAG repeat length. 

As well as exhibiting altered neurodevelopment, HD iPSC-derived neuronal cultures have been associated with impaired neuronal maturation and electrophysiological activity, especially in juvenile HD lines. While the resting membrane potential (RMP) of normal iPSC-derived cortical neurons became hyperpolarised with time in culture, indicative of neuronal maturation, the RMPs of juvenile HD iPSC-derived cortical neurons remained depolarised [63]. It is important to note that both normal and HD iPSC-derived cortical neurons [63] and striatal neurons [62] still displayed depolarised RMPs reflective of immature neurons. Nonetheless, the altered expression of genes involved in electrophysiological activity has been reported in juvenile HD iPSC-derived neurons [47] and cortical neurons [63], with HD iPSC-derived neuronal cultures from a 180 CAG repeat line failing to produce action potentials altogether [45]. Store-operated calcium channel (SOC)-mediated calcium dysfunction has been observed in HD iPSC-derived neuronal cultures with juvenile [28] and adult-onset [25,27] CAG repeat lengths, implicating altered functionality in both juvenile and adult-onset HD. In HD 60 CAG iPSC-derived neuronal cultures, calcium dyshomeostasis was exacerbated by glutamate pulses and eventually led to cell death [45], providing support for the excitotoxicity hypothesis of HD. 

However, there are conflicting reports on the expression of calcium signalling genes in HD iPSC-derived neuronal cultures, with Mattis et al. reporting no difference in the expression of the N-methyl-D-aspartate (NMDA) receptor subunit *GRIN2B* [41], and the HD iPSC Consortium reporting reduced expression of *GRIN2B* as well as other calcium signalling genes [43]. Given that two of the three lines used by Mattis et al. [41] were the same two lines used by the HD iPSC Consortium [43], these discrepancies could implicate differences in neuronal yield, culture protocols, and experimental procedures as sources of variability that may influence the manifestation of disease-associated phenotypes.

#### 2.2.4. Cell Stress and Degenerative Phenotypes Observed in Huntington’s Disease Neurons Derived from Induced Pluripotent Stem Cells

The nature of cell stress-associated phenotypes varied between juvenile and adult-onset HD models. While both juvenile [55] and adult-onset [30] HD iPSC-derived neuronal cultures exhibited decreased expression of oxidative stress response genes or proteins, the former demonstrated increased expression of DNA damage response genes and proteins [55], while the latter demonstrated increased or unchanged expression of these factors [30]. Juvenile HD iPSC-derived neuronal cultures also exhibited mitochondrial dysfunction-associated phenotypes, including decreased mitochondrial membrane potential [64], increased production of mitochondrial ROS [64], increased fragmentation of neurite mitochondria [64], and mitochondrial respiration deficits [36]. Decreased ATP levels were observed in a CAG repeat length-dependent manner [36,64]. However, this reduction was also present in HD iPSCs, iPSC-derived NPCs, and iPSC-derived neuronal cultures, indicating that this phenotype was not neuron-specific [36,64]. The mitochondrial phenotypes were corrected using an inhibitor of DRP1-mediated mitochondrial fission [64], p53 knockdown [64], or isogenic correction of the CAG expansion [68], supporting the link between mitochondrial dysfunction and increased cell death in HD. Importantly, mitochondrial trafficking impairments were observed in adult-onset HD iPSCs, manifesting as reduced neurite mitochondrial densities and an increased vulnerability to further reduction following proteasome inhibition [26]. 

Juvenile HD iPSC-derived neuronal cultures exhibited increased caspase-3/7 activity, activation of apoptotic mechanisms, and cell death, especially in cell lines with greater than 100 CAG repeats [45,55,64]. In contrast, no difference in caspase activity or cell death was observed in adult-onset HD iPSC-derived neuronal cultures unless they were exposed to exogenous cell stressors [30,45]. As hypothesised previously, this may indicate that juvenile HD iPSC-derived neuronal cultures are more susceptible to cell stress under standard culture conditions than the adult-onset HD counterparts. Again, it is unknown whether this heightened vulnerability is merely residual from iPSCs and the reprogramming process itself, or whether it reflects a heightened vulnerability to cell stress in neuronal cultures. Notably, adult-onset HD iPSC-derived neuronal cultures exhibited increased macroautophagy [24], increased autophagosome activity [25], and increased lysosomal activity [25]. These phenotypes are consistent with the removal of dysfunctional machinery and improperly processed proteins, suggesting that adult-onset HD iPSC-derived neuronal cultures activated these protective pathways in response to early pathogenic events [79,80,81,82]. Interestingly, while Joshi et al. observed increased resistance to manganese toxicity in HD iPSCs and iPSC-derived cortical NPCs, there was no difference in manganese toxicity in HD iPSC-derived cortical neurons [40]. Furthermore, HD iPSC-derived early midbrain neurons exhibited increased sensitivity to manganese [40], highlighting the importance of cell type-specific phenotypes when interpreting the results of HD iPSC reprogramming studies. 

#### 2.2.5. Genetic Correction and the Validation of Huntington’s Disease Phenotypes Using Neurons Derived from Induced Pluripotent Stem Cells Using Isogenic Controls

A significant advantage of modelling monogenetic disorders is the ability to genetically correct the disease-causative mutation to generate isogenic control lines from the same individual. While initially time-consuming and technically challenging, recent advances in gene-editing technologies, such as the clustered regularly interspaced short palindromic repeats (CRISPR)/Cas9 system, have made the generation of isogenic lines an attractive approach for disease modelling [83]. Successful correction of the HD mutation in iPSC lines from individuals with CAG repeat lengths of 72 [54,61], 92 [84], 109 [85], and 180 [68] have been performed. However, gene editing technologies are not without limitations and can confer a substantial risk of off-target effects as they rely on the host cell’s ability to repair DNA breaks induced by the gene-editing mechanisms. HD iPSCs exhibit alterations in pathways required for the repair of DNA break-induced damage [30,38,49,55,65]. As well as affecting the success of genetic correction, these altered repair mechanisms may mask or display false phenotypes in gene-corrected isogenic control lines. Thus, using cell lines from affected and unaffected family members may be a more appropriate approach.

Interestingly, Xu et al. reported that the gene expression differences between normal and HD in an iPSC model were absent when compared between HD and isogenic controls [65]. The authors hypothesised that the observed gene expression differences between normal and HD were a result of inter-individual genetic variation, and not the disease-causative mutation itself. Importantly, Xu et al. reported that, unlike gene expression, the phenotypic differences observed in HD cells were corrected by the isogenic controls [65]. Similarly, Lopes et al. observed that genetic correction of a 72 CAG HD iPSC line improved basal respiration and reduced mitochondrial ROS in the NPCs yet did not restore the altered expression of *PGC1α* or *TFAM* [59]. Malankhanova et al. used CRISPR/Cas9 gene editing to induce a 69 CAG repeat expansion in healthy human embryonic fibroblasts which were then reprogrammed into iPSCs and differentiated into MSNs alongside the unedited control and an HD iPSC line with 47 repeats [32]. Importantly, they observed that the impairments in neural rosette formation and susceptibility to BDNF withdrawal present in the genetically induced HD iPSC-derived neural cells were similar to those observed in the 47 CAG repeat line and were absent in the original lines from which the 69 CAG repeat line was generated. This work highlights the potential use of genetically modified iPSC lines to study HD-specific phenotypes, although care should be taking when extrapolating findings from genetically modified iPSC lines to the wider HD population.

### 2.3. Challenges of Pluripotent Stem Cell Models of Huntington’s Disease

While the indefinite expandability of PSCs is an attractive feature of this cell type, there is a strong link between repeated PSC passaging and the accumulation of genomic abnormalities [86,87,88,89,90,91]. Up to 13% of published human PSC lines contain abnormal karyotypes [86]. Although up to 40% of primary human neurons may exhibit at least one de novo copy number variation, the clonal nature of iPSC colonies is likely to result in a greater pattern of variation and mosaicism [92]. With increased passaging also comes an increased risk of genomic instability, thus even commercially available PSC lines with normal karyotypes may accumulate abnormalities over time [87,89,90]. Karyotype abnormalities that PSC lines can easily gain through reprogramming or extensive subculturing might impact on their differentiation potential and could mask or induce disease phenotypes [86]. The use of mouse embryonic cell feeder layers during iPSC propagation reduces the genomic instability that comes with enzymatic dissociation and passaging [93]. However, the dependence on animal cell co-culture substantially limits downstream clinical translation due to the highly variable and competitive nature of iPSC proliferation, with clonal iPSC expansion selecting for healthy over unhealthy iPSCs [94]. This presents a considerable challenge in disease modelling, as disease-associated phenotypes present in the host cells may be diluted out of PSC cultures. 

The appropriateness of HD iPSC reprogramming models depends on the suitability of iPSCs in disease modelling and the relevance of screening for potential disease modifiers in what is essentially a neurodevelopmental model. To date, no HD iPSC lines have been generated using DNA-free techniques. Although episomal delivery of the reprogramming factors has been utilised more frequently in recent years to generate HD iPSCs, studies continue to use the better-characterised HD iPSC lines generated from viral reprogramming techniques. The reprogramming method itself may also alter the phenotype of iPSCs, with Tidball et al. reporting somatic instability and differential activation of apoptotic pathway factors when iPSCs were generated with [38] or without [49] shRNA against p53 to increase reprogramming efficiencies. Even if the CAG repeat expansion affects the generation of HD iPSCs compared to normal, this has little clinical or physiological relevance. Alterations in the generation of HD iPSCs, whether related to the CAG expansion or inter-individual variability, could lead to the persistence of what appears to be a disease-associated phenotype in iPSC-derived cells, yet is more reflective of the intrinsic variability of pluripotency reprogramming itself. 

One of the main caveats of iPSC generation for downstream disease modelling applications is the epigenetic reset required for the acquisition of pluripotency [95]. Although iPSCs can at least partially maintain the epigenetic memory of the parent cell type at the DNA methylation level [96,97], this memory is removed by continued passaging [98]. Comparable to hESCs, iPSCs are thought to reflect the first-trimester stage of development, even when obtained from elderly donors and terminally differentiated into mature cells [99]. Miller et al. confirmed that age-associated phenotypes present in fibroblasts from aged donors such as reduced telomere length, mitochondrial dysfunction, impaired DNA damage response, loss of heterochromatin, nuclear alterations, and increased cell senescence, were reset to a healthy embryonic-like state during iPSC reprogramming [100]. Studer et al. proposed that these rejuvenated iPSCs resulted from the preferential expansion of a minor population of relatively healthy fibroblasts [95], as fibroblast cultures are notoriously heterogeneous [92,101]. As such, iPSC-derived cells from aged donors and individuals with neurodegenerative disorders may have a limited capacity for modelling disease-associated phenotypes dependent on the accumulation of age-related pathologies. The role of accumulated age-related pathologies in HD neuropathogenesis is unclear. However, the heterogenetic clinical presentation, age of symptom onset, and disease severity in HD support the presence of disease-modifiers beyond the initial HD-causative CAG expansion [102,103,104,105].

Mollica et al. confirmed that the altered expression of DNA damage response genes in HD fibroblasts was normalised in HD iPSCs [29,106]. Given the similarity of hESCs and iPSCs, and the decades-long delay before HD symptom onset, it is not surprising that both HD hESCs and iPSCs do not recapitulate the neurodegeneration that occurs during the symptomatic stages of HD. Even in juvenile HD iPSC-derived neuronal cultures, a neurodegenerative phenotype is rarely reported under standard culture conditions. This presents a challenge when using HD iPSC-derived neuronal cultures to screen for potential therapeutics to protect against degeneration. Only Guo et al. have investigated the potential of therapeutic interventions in HD iPSC-derived neuronal cultures [64]. As the HD iPSCs were from a juvenile HD line with a large (100 CAG) repeat length and symptom onset at two years old, it could be reasoned that this line exhibited a particularly severe disease phenotype. Thus, the therapeutic benefit of the trialled interventions may have limited applicability to the majority of HD cases. 

Multiple methods have been employed to encourage degeneration in HD iPSC-derived neuronal cultures. BDNF withdrawal [34,35,41,42,45,65], artificially-induced oxidative stress [30,32], and excitotoxic challenges [45] result in caspase activation [34,35,45] and eventual cell death [32,35,41,45,65]. Using this system, studies have investigated the effects of therapeutic agents in reducing or rescuing HD iPSC-derived neuronal cultures from induced cell stress, identifying ATM inhibition [65], G-protein signalling receptor knockdown [35], TrkB receptor agonist antibodies [41], calcium chelators [41], MAPK signalling inhibitors [41], NMDA and AMPA receptor antagonism [41], increased BDNF concentration [45], and PPARδ activation [42] as neuroprotective. Although the cell stressors these compounds are protective against may also be present in the HD brain, the acute nature of these experiments in HD iPSC-derived neuronal cultures is unlikely to reflect the accumulation of cell stress that would occur over decades in those with HD. 

Similarly, while artificial aging using progerin has been considered as an alternative method of encouraging age-related pathologies in iPSC models of neurodegenerative disorders [22], the physiological relevance of this technique is questionable [100]. Machiela et al. demonstrated that adeno-associated virus (AAV) transduction of progerin in iPSC-derived NPCs resulted in increased damage in differentiated HD cells but not controls, although both the differentiated control and HD cells exhibited reduced dendritic lengths and similar caspase activation when transduced with progerin as iPSC-derived NPCs [31]. As two of the three HD iPSC lines investigated by Machiela et al. were from juvenile cases of HD, the specificity of artificially induced aging to model the changes that occur before adult-onset HD are called into question. Accordingly, the most appropriate use for HD iPSC models in drug screening applications may be to investigate the effects of therapeutic interventions targeting phenotypes observed during the pre-degenerative stages in the absence of cell stress. Due to the difficulty in obtaining a disease-associated phenotype in adult-onset HD lines under standard culture conditions, the use of HD iPSC models in drug screening applications may be predominantly limited to juvenile HD. 

## 3. Direct Cell Reprogramming to Model Huntington’s Disease

### 3.1. Induced Neuron Reprogramming as an Alternative to Induced Pluripotent Stem Cells to Study Huntington’s Disease Neuropathogensis

A significant technical advancement in the field of cell reprogramming was the discovery that somatic cells could be directly converted into neurons, thus avoiding the genomic instability and epigenetic reset associated with the generation and propagation of iPSCs. Neurons generated using direct-to-induced neuron (iN) reprogramming retain an age-associated transcriptional profile and demonstrate age-related mitochondrial defects that are consistent with the age of the donor cell [107,108]. Although the importance of retaining age-related pathologies in cell reprogramming models of HD is unclear, the limited capacity to generate HD iPSC-derived and hESC-derived neurons that spontaneously exhibit the hallmark features of HD make direct cell reprogramming an attractive alternative for HD modelling and downstream drug screening applications. 

To date, iNs have been generated from fibroblasts obtained from individuals with adult-onset [109,110] and juvenile [111] HD using REST inhibition via PTBP1 knockdown [111] or miR-9/9*-124 in combination with neuronal [109] or striatal lineage [110] transcription factors (Table 6). Although variable between studies, the neuronal yields were similar in normal and HD cultures [109,110,111]. Both Liu et al. [111] and Victor et al. [110] reported the generation of predominantly DARPP32+ neurons. Unlike the latter study, Liu et al. reported increased DARPP32+ yields in HD as well as abnormal dendritic branching and neurite degeneration [111]. Both juvenile [111] and adult-onset HD [110] exhibited increased neuron loss over time. Liu et al. reported apoptotic cell death and the loss of GABAergic neurons, consistent with preferential degeneration of striatal neurons in HD [111]. Notably, both normal and HD neuron yields decreased with time in culture, indicating that neuronal loss was partly reflective of the model itself [110,111]. 

Victor et al. observed altered expression of neuronal and synaptic genes in HD-derived iNs [110]. Altered expression of calcium signalling genes was observed in iNs from adult-onset HD [110]. HD iNs also demonstrated increased expression of synaptic genes, including GABA and AMPA receptor subunit genes, contradicting the observations of HD iPSC-derived neuronal cultures [43]. Interestingly, adult-onset HD iNs exhibited higher frequencies of multiple action potentials compared to normal iNs [110]. HD iNs were also more excitable than normal iNs [110], consistent with the excitotoxicity observed during the early stages of HD in animal models [112,113,114,115]. Unlike HD iPSC-derived neurons, the RMPs of HD and normal iNs were similar, and both HD and normal iNs had RMPs more reflective of mature neurons than iPSC-derived neurons [110]. HD iNs also demonstrated reduced expression of *NTRK2* encoding the TrkB receptor, consistent with the reduced expression of *NTRK2* and the TrkB receptor observed in HD post-mortem brain tissue [74,116,117,118,119,120,121]. Victor et al. reported phenotypes consistent with HD progression, including mitochondrial dysfunction, increased DNA damage, increased ROS, and lysosomal impairments [110]. Notably, these phenotypes were observed in adult-onset HD iNs [110], having been rarely reported in adult-onset HD iPSC-derived neuronal cultures. This indicates that direct reprogramming and the resulting maintenance of age-related phenotypes that are erased by iPSC reprogramming may be more adequate for modelling adult-onset HD.

Most importantly, both Liu et al. [111] and Victor et al. [110] reported the presence of nuclear and cytoplasmic HTT aggregates in HD iNs under standard culture conditions, with cytoplasmic inclusions present in 6–10% of HD iNs and nuclear inclusions present in 4–10% of HD iNs, consistent with HD human post-mortem brain tissue [122,123]. In contrast, HTT aggregates were only observed in HD iPSC-derived neuronal cultures in the presence of proteasome inhibition [25,58] or when transplanted into the QA lesioned rodent model of HD as neurospheres [56]. Victor et al. demonstrated that the same HD lines that exhibited HTT aggregates in iNs failed to generate aggregates when reprogrammed into iPSCs first, indicating that the formation of aggregates is dependent on the actual reprogramming system [110]. Victor et al. also demonstrated the neuroprotective benefits of ATM inhibition against cell death in HD iNs both in the absence and presence of artificially-induced cell stress [110]. Collectively, these studies highlight the advantages of direct reprogramming for generating HD iNs that spontaneously exhibit disease-associated phenotypes, thus could be used to screen for phenotype-modifying treatments.

### 3.2. Considerations for the Use of Directly Induced Neuron Models of Huntington’s Disease

It is important to note that neurons generated from direct-to-iN reprogramming have not followed the successive stages of neurodevelopment that are followed in vivo or PSC reprogramming. Instead, the acquisition of a neuronal identity is dependent on the overriding of the starting cell types transcriptional profile, often via the overexpression of pro-neuronal transcription factors, the effectiveness of which depends on the starting cell type [124,125]. While intermediate cell types are reported during the conversion of somatic cells into iNs, these cell types represent unique intermediates incongruent with the transcriptional profile of both the starting and target cell type [124]. Thus, direct-to-iN reprogramming may not be appropriate for modelling neurodevelopmental conditions or conditions with a neurodevelopmental component, such as HD [126]. HD iN studies are limited in their ability to model disease-associated mechanisms that may occur during neurodevelopment and neuronal maturation and may not be suitable for investigating treatments targeting neurodevelopmental alterations and preventing the progression of HD before the onset of symptoms. Furthermore, all current HD iN studies have used lentiviral delivery of the reprogramming factors and have depended upon REST inhibition to induce neuronal conversion. The latter feature is particularly concerning for modelling HD due to the inability of mHTT to suppress REST expression in the HD brain [71,75,127]. Although some disease-associated phenotypes were observed in HD iNs the presence of miR-9/9*-124-mediated REST inhibition, the increased cell death in HD iNs following the removal of this REST inhibition may have been atypically exacerbated by the sudden increase in REST activity in HD iNs [110]. As the removal of miR-9/9*-124 from the reprogramming factors drastically reduced neuronal yield, the suitability of iNs in HD modelling may depend on future advancements in direct cell reprogramming to generate high neuronal yields without inhibitors of REST.

As somatic cells are directly reprogramming into post-mitotic iNs without passing through an expandable progenitor stage, the output capacity of direct-to-iN reprogramming is limited by the size of the starting cell population. Thus, starting cell populations from a greater number of donors and increased numbers of biological replicates are needed in direct-to-iN reprogramming to obtain sufficient numbers of iNs. While often regarded as a drawback of direct reprogramming, the heterogeneity of this technique provides the opportunity to develop a powerful tool for data analysis that better represents the inter-individual variability and mosaicism present in human populations [128]. Interestingly, the retention of inter-individual variability in vitro may be important for recapitulating intrinsic human variability and could hold the potential to generate a powerful model of the heterogeneous nature of human diseases. Due to the epigenetic reset caused by iPSC reprogramming, subtle disease phenotypes and modifiers may be diluted out or erased from HD iPSC-derived cultures [95,100]. Coupled with the limited number of different donor cell lines typically used in iPSC reprogramming studies, this makes it difficult to determine which phenotypes are disease-associated and which are a product of clonal variation and the cell culture conditions. The requirement for high numbers of different donor cell lines in direct reprogramming in combination with the likely retention of age-accumulated and subtle disease-associated phenotypes could represent a more appropriate system for investigating the contribution of factors beyond the CAG expansion to the HD phenotype. Genetic, epigenetic, and environmental modifiers may all play a role in the variability of the age of symptom onset, progression, and severity of HD beyond the length of the CAG repeat expansion between individuals with HD [129,130]. 

### 3.3. Induced Neural Precursor Cells as an Alternative Method of Cell Reprogramming

Despite the substantially higher conversion efficiencies, shorter time frames, and retention of epigenetic signatures in direct-to-iN reprogramming, iPSC reprogramming prevails as the preferred technique for disease modelling, drug screening, and cell replacement therapy research. This may be predominantly due to the generation of defined NPC intermediates from iPSCs. Direct-to-induced neural precursor cell (iNP) reprogramming represents an attractive technology for generating NPCs without the genetic instability and limited clinical translatability of iPSCs. Importantly, iNPs can also be generated directly from human fibroblasts without the inclusion of pluripotency factors, with similar efficiencies, experiment durations, expression of NPC markers, differentiation capacity, and functional activity as pluripotency factor-derived iNPs. 

In 2012, we reported the generation of iNPs from adult human fibroblasts using *SOX2* and *PAX6* cDNA plasmids or SOX2 and PAX6 recombinant protein expression [131]. Plasmid-derived iNPs expressed SOX2, PAX6, ASCL1, and NGN2 mRNA and protein, as well as the neural markers *SOX1*, *HOXB9*, *NKX6.1*, and *SIX3*. iNPs differentiated into both GABAergic and glutamatergic neurons and a small proportion of astrocytes within 30 days and exhibited neuronal functionality. However, the reprogramming efficiency was low (0.05%), and the duration was 85 days long [131]. In 2018, our research group reported the generation of iNPs from adult human fibroblasts with a higher reprogramming efficiency (0.3%) and shorter time frame (21–28 days) following the transient expression of chemically-modified mRNA (cmRNA) encoding *SOX2* and *PAX6* [132]. As well as neural and telencephalic progenitor markers, the iNPs expressed LGE transcription factors GSX2 and MEIS2. The iNPs differentiated into predominantly glutamatergic or DARPP32+ neurons depending on the protocol and exhibited immature functionality [132]. This cmRNA-mediated direct-to-iNP reprogramming technique represents a novel platform for generating iNPs and neurons from adult human fibroblasts. 

### 3.4. Induced Neural Precursor Cell-Derived Neuronal Models of Huntington’s Disease

The direct generation of iNPs from HD somatic cells may be the key to addressing the limitations of both iPSC and iN models of HD. Hou et al. provided proof-of-principle evidence that adult-onset HD fibroblasts could be reprogrammed via the lentiviral delivery of six or seven reprogramming factors to generate embryonic-like iNPs that differentiated into neurons, astrocytes, and oligodendrocytes [133] (Table 7). The increased expression of the DNA damage response marker γH2AX present in HD fibroblasts was not corrected by the reprogramming or differentiation processes [133]. In contrast, the authors needed oxidative stress to induce the increased expression of γH2AX in HD iPSC-derived neurons [30]. This suggests that, like direct-to-iN reprogramming [110] and unlike iPSC reprogramming [29], direct-to-iNP reprogramming retains the pathologies present in HD fibroblasts when reprogrammed. Although the expression of γH2AX in normal fibroblasts and iNPs was similar, the expression of γH2AX increased upon reprogramming HD-derived HDFs to iNPs [133]. Furthermore, both normal and HD iNPs exhibited increased expression of γH2AX upon differentiation [133]. This suggests that while the differentiation of iNPs into neurons increased the cell stress response in both normal and HD, the HD fibroblasts were also susceptible during the reprogramming to iNPs [133]. 

Given the number of transcription factors and the lentiviral delivery system used by Hou et al. [133], it is unclear whether the trends of γH2AX expression over time in normal and HD were reflective of a disease phenotype, or whether they were a result of the reprogramming process itself. Nonetheless, Hou et al. used this system to investigate the therapeutic effects of adenosine receptor inhibition on reducing the expression of γH2AX in HD fibroblasts, iNPs, and differentiated cells [133]. Unlike their original study [30], the authors verified the neuroprotective benefits of adenosine receptor inhibition in the absence of artificially-induced cell stress [133]. While adenosine receptor inhibition did not affect the expression of γH2AX in normal fibroblasts, iNPs, or differentiated cells, it reduced the expression of γH2AX in HD fibroblasts and iNP-derived differentiated cells to normal [133]. Although Hou et al. did not report the HD pathologies specifically in neurons, they provided valuable evidence that direct-to-iNP reprogramming may be used to screen for potential HD therapeutics in the absence of artificially-induced cell stress. This highlights the potential of direct-to-iNP reprogramming to generate a non-rejuvenated model of HD that can be used to investigate HD during neuronal differentiation.

We recently established the first direct-to-iNP striatal reprogramming model of HD and demonstrated for direct reprogramming of HD patient-derived fibroblasts with clinically relevant CAG repeat lengths using cmRNA generated iNPs that different into DARPP32+ neurons [134]. Both normal and HD iNPs expressed striatal lineage markers (GSX2, ASCL1, MEIS2) and differentiated into comparatively high yields of neurons and striatal-phenotype neurons. Furthermore, the transcriptional profiles of normal and HD reprogrammed and differentiated cells were similar, suggesting a differential role for impaired neurodevelopment in juvenile HD versus adult-onset HD. Importantly, we observed ubiquitinated HTT aggregates in differentiated cells from the 57 CAG HD line, a finding consistent with HD iNs [110] and early-stage HD post-mortem human brain tissue [135]. The detection of ubiquitinated HTT in direct-to-iNP-derived differentiated HD cells indicates that our direct-to-iNP reprogramming HD model conserves HD-associated phenotypes that are typically erased by iPSC reprogramming. 

Significantly, we observed that the HD iNP-derived neurons exhibited phenotypes that were consistent with impaired neuronal maturation, including smaller cell soma areas, shorter neurite lengths, and reduced neurite branching. These morphological differences in the HD iNP-derived neurons was consistent with juvenile HD iPSC-derived neurons [55,63,64] and juvenile HD iNs [111]. Furthermore, the HD iNP-derived differentiated cells demonstrated significantly more depolarised RMPs compared to normal iNP-derived differentiated cells, consistent with impaired neuronal maturation and altered functional activity. Our findings also provide support for the link between altered BDNF activity and HD [136,137], as we observed reduced BDNF protein expression in HD lines that correlated with measures of HD severity, including earlier age of HD symptom onset and longer CAG repeat length expansions. We propose that direct-to-iNP reprogramming generates a more clinically relevant in vitro model of HD than iPSC reprogramming models of HD, which are frequently generated using juvenile HD lines and erase important age-related phenotypes that may be key factors in the more common adult-onset form of HD. Furthermore, the presence of an intermediate progenitor stage in direct-to-iNP reprogramming provides an important platform for studies of neuronal maturation and to identify and test novel targets for HD therapeutic intervention.

## 4. The Use of Cell Reprogramming for Disease Modelling and Recent Advancements in Cell Reprogramming Technologies

### 4.1. The Use of Cell Reprogramming to Study Cell-Autonomous and Non-Cell-Autonomous Mechanisms of Huntington’s Disease Neuropathogenesis

The critical advantage of cell reprogramming over other cellular models is the ability to study disease-associated mechanisms in the cell type predominantly affected by the disease, such as MSNs in HD. The generation of purely neuronal populations using cell reprogramming is not practically feasible, nor is it physiologically representative of in vivo environment. For instance, MSNs interact with glia and receive neurotrophic support from cortical and dopaminergic neurons in vivo [138,139,140,141]. Although in vitro neuronal differentiation is not yet fully disciplined and the production of multiple cell types is usually an off-target effect, careful refinement of the protocols for generating both relatively pure and deliberately mixed neuronal and non-neuronal cultures could be an invaluable tool for studying both the cell-autonomous and non-cell-autonomous mechanisms of disease. However, the generation of mixed differentiated cell cultures presents a technical challenge for cell reprogramming disease models, as the presence of other cell types in the population may lead to the under-representation or over-representation of disease-associated phenotypes in population studies. 

### 4.2. The Generation of Mixed Neuronal and Glial Cell Cultured from Directly Reprogrammed Induced Neural Precursor Cells for Modelling Huntington’s Disease

While MSNs are preferentially affected in HD, astrocytes [142,143,144,145,146], microglia [147,148], and oligodendrocytes have all been implicated in HD neuropathogenesis, especially during the later disease stages. For example, iPSC-derived astrocytes from HD cell lines demonstrated a disease-associated vacuolisation phenotype [76] and electrophysiological impairments [78]. Merienne et al. reported that in a mouse model of HD, the upregulated genes were highly represented in glial cells, whereas the downregulated genes were highly represented in neuronal cells. This suggests that transcriptional changes in HD might be cell-type specific, although the authors did not report whether these changes occurred cell-autonomously or non-cell-autonomously. As such, transcriptional changes in glial cells could mask transcriptional changes in neuronal cells, prompting the need for analysis technologies that can distinguish neurons from glia [149]. The emergence of both neurons and glia in differentiated cultures from the same HD cell line could represent a more physiologically relevant system for assessing the effects of glial cells on HD pathogenesis than traditional co-culture methods that use rodent glial cells [110,150].

### 4.3. The Challenge of Using Small Molecules and Small Molecule Inhibitors to Enhance Nuronal Yield and Functionality for Huntington’s Disease Modelling Applications

A significant limitation of traditional cellular models of HD, such as lymphoblasts and fibroblasts obtained from individuals with HD, is that they do not allow for the study of disease neuropathogenesis in the cell type that is predominantly affected by the disease. With the advent of cell reprogramming, HD neurons can be generated in vitro from easily obtainable somatic cell types that carry the genetic background of HD. hESC, iPSC, and direct reprogramming protocols produce highly variable neuronal yields, being influenced by protocol differences, culture duration, inter-individual variability, experimental variability, and numerous other factors. In cell reprogramming models of HD, neuronal yields range from less than 10% TUJ1+ to more than 90% TUJ1+ across iPSC and direct reprogramming studies. 

Due to the difficulty of reprogramming adult human HDFs, many cell reprogramming protocols incorporate small molecules and small molecule inhibitors to enhance reprogramming efficiencies and neuronal fate conversion [151,152,153,154,155,156]. Careful consideration must be given to the possible effects of small molecules and small molecule inhibitors in masking, exacerbating, or even rescuing disease-associated phenotypes. The inclusion of the neurogenesis inducer isoxazole-9 may significantly improve the neuronal yield [155]. However, isoxazole-9 reversed the developmental alterations in MSN-like neurons derived from HD iPSCs [43]. Furthermore, HDAC inhibitors are often used in the differentiation of HD iPSCs, despite the reported neuroprotective effects of HDAC inhibitors in animal models of HD and human clinical trials [37,41,43,45,157]. Similarly, ROCK inhibitors are frequently included in cell reprogramming to protect cultures against cell death [158,159]. This may explain why the manipulation of cell culture conditions is often required to induce neurodegeneration in HD reprogrammed neurons and overcome the protective effects of ROCK inhibitors.

The potential disease-modifying effects of small molecules and small molecule inhibitors present a challenge when optimising cell reprogramming protocols for disease modelling. JNK inhibitors [156], PKC inhibitors [156], and GSK3β inhibitors [152,153,155,156] have all demonstrated beneficial effects in cell reprogramming. However, many of these inhibitors target signalling factors downstream of the TrkB and p75 receptors [160,161]. Abnormal signalling of BDNF via the TrkB and p75 receptors is strongly implicated in HD neuropathogenesis. Thus, while including small molecule inhibitors may enhance the neuronal yields obtained in this model, these factors could also mask important disease phenotypes that affect growth factor and receptor signalling. 

Irrespectively, small molecules and small molecule inhibitors may be essential in the conversion of adult HDFs into iNPs and the subsequent generation of functionally mature neurons [162]. For example, cyclic adenosine monophosphate (cAMP) and cAMP agonists are widely used in iPSC and direct reprogramming protocols to promote the generation of functional neurons [150,154,155,156] and are included in many HD iPSC models [25,37,41,43,45,64]. However, cAMP signalling may also be reduced in HD [163,164]. As such, promoting cAMP signalling using small molecules could have neuroprotective effects that interfere with this potential phenotype in HD. 

Consequently, there is a difficult trade-off between including factors that may enhance reprogramming and differentiation and excluding factors that may potentially alter the HD phenotype. It may be most appropriate to investigate the effects of the underlying culture conditions on the generation of high yields of normal and HD iNP-derived neurons before incorporating small molecules that could interfere with crucial cell signalling pathways implicated in HD neuropathogenesis. For example, altering cell plating density, the basement membrane upon which cells are grown, or culturing cells in a hypoxic workstation may be sufficient to improve neuronal yield and functionality without altering the HD phenotype or the pathways that contribute to HD neuropathogenesis. 

### 4.4. The Generation of Multiple Neuronal Subtypes from Directly Reprogrammed Induced Neural Precursor Cells for Modelling Huntington’s Disease

The refinement of protocols to generate disease-specific and clinically relevant cell types is one of the most attractive features of cell reprogramming for disease modelling. In vivo, highly regulated extrinsic cell signalling cues, transcriptional regulators, and epigenetic modifiers govern neuronal subtype identity. Harnessing the knowledge of how the brain microenvironment gives rise to distinctly different neuronal subtypes in vivo has led to the development of in vitro differentiation protocols that preferentially generate one neuronal subtype over another. By manipulating the environmental conditions of differentiating cells using small molecules and growth factors, NPCs can be ventralised and differentiated into other neuronal subtypes, including striatal MSN-like neurons. The proportion of MSN-like neurons is highly variable in iPSC reprogramming models of HD, from 5–80% DARPP32+/TUJ1+ (Table 5). Direct reprogramming models of HD tend to generate higher proportions of DARPP32+ neurons, from 55–70% DARPP32+/TUJ1+ (Table 6 and Table 7). Although MSNs do not exist in isolation in vivo, the generation of an almost entirely MSN-like neuronal population makes it easier to study cell-autonomous effects of the HD mutation on the cell type predominantly affected by the disease.

Even gold-standard MSN differentiation protocols do not generate neuronal populations consisting entirely of DARPP32+ neurons. Furthermore, there is considerable variability between individual cell lines in the generation of subtype-specific neurons, especially in hESC and iPSC reprogramming studies [165]. By default, human reprogrammed NPCs take on a telencephalic identity that leads to the preferential generation of dorsal forebrain glutamatergic and GABAergic neurons in the absence of regionally-specific patterning cues [166]. Apart from MSNs, cortical neurons undergo the most considerable dysfunction and degeneration in HD, with up to 30% of cortical neurons lost in end-stage disease [167,168]. Furthermore, alterations in the cortico-striatal tracts between cortical neurons and MSNs in HD are thought to be a substantial contributor to excitotoxicity and the BDNF deficit hypothesis of HD neuropathogenesis [113,114,115,139,169,170,171,172,173,174]. The presence of both cortical and striatal neurons in the differentiated cultures may provide a novel system for studying cortico-striatal tract abnormalities in HD patient-derived cells, without the questionable physiological relevance of using healthy rodent or fetal neuron co-culture systems [175]. By taking advantage of the default differentiation of human reprogrammed cells into cortical neurons, the careful manipulation of ventral patterning factors in the differentiation medium could be one method of obtaining mixed cultures for modelling cortico-striatal tract pathologies in HD, especially if combined with microfluidic chamber systems that can elegantly display these tracts in isolation for imaging studies [117,119]. For example, Virlogeux et al. reconstituted the cortico-striatal network in vitro using primary neurons from a rodent model of HD to identify presynaptic, synaptic, and post-synaptic deficits in cortico-striatal tracts in HD rodents [176].

### 4.5. The Inclusion of Three-Dimensional Culture Systems to Generate Functionally Mature Striatal Neurons from Reprogrammed Cells

Like the production of high neuronal yields, a substantial challenge faced in the generation of neurons using cell reprogramming is the difficulty in obtaining functionally mature neurons representative of those that exist in humans in vivo. Immature neuronal functionality does not necessarily correlate with reprogrammed neurons being equivalent to fetal neurons at a transcriptional level, as Victor et al. reported a similar transcriptomic profile in directly-induced MSNs and micro-dissected MSNs from adult human brain tissue [175]. However, both iPSC-derived neurons and iNs are believed to be more functionally similar to fetal neurons than to adult neurons [177,178]. This is of particular concern when studying the effects of cell stress on reprogrammed neurons, as terminally differentiated mature neurons have a greater resistance to cytotoxic stimuli and cell stressors than immature neurons [179]. 

Neurons form extensive networks and interact with a multitude of neuronal and non-neuronal cell types in vivo and depend upon the complex microenvironment created by these networks and interactions for metabolic, functional, and structural support [180]. The influence of the local microenvironment on neuronal function may have a significant role in neurological dysfunction. Advancements in cell reprogramming techniques seek to recreate these complex microenvironments in vitro using three-dimensional culture systems. Recently, Adil et al. demonstrated the generation of striatal progenitors from human PSCs within a fully-defined and scalable three-dimensional hydrogel system [181]. Even when these three-dimensional-derived striatal progenitors were matured in two-dimensional systems, they generated high yields of mature neurons (78% MAP2+; 55% DARPP32+). The majority of three-dimensional-derived neurons demonstrated spontaneous action potentials by day 60 of differentiation, whereas two-dimensional-derived neurons required 90 days of differentiation to demonstrate similar functionality. 

### 4.6. The Generation of Huntington’s Disease Organoids for Modelling the Non-Cell-Autonomous Mechanisms Driving Medium Spiny Neuron Dysfunction

Even conventional co-culture systems and three-dimensional matrices may be limited in their capacity to resemble the in vivo cellular microenvironment of the human brain and the complex cell-to-cell connections that drive neuronal function in vivo [182,183,184]. This is especially important when studying disease-associated events that may occur during neurodevelopment, a process that is heavily influenced by external cues provided by the reprogramming medium in traditional cell reprogramming models. A promising advancement in cell reprogramming is the generation ‘mini-brain’ organoids or brain region-specific spheroids [185,186]. The self-assembly of PSCs into multi-layered epithelial structures and progenitor zones may generate a model that is more representative of the spatiotemporal cellular interactions and complex microenvironment of the brain in vivo than traditional in vitro culture systems. While organoids currently lack the environmental cues to develop into fully functional and mature organs, they can give rise to functionally mature neurons [187]. Although there have been no reports to date of organoids developed from direct reprogramming technologies, the presence of a progenitor stage in direct-to-iNP reprogramming highlights this technology as a potential method for generating organoids that bypass many of the limitations associated with pluripotency. 

Currently, only one study has reported the generation of organoids from HD iPSCs, with Conforti et al. using juvenile HD cell lines to generate organoids containing predominantly cortical neurons [47]. This model demonstrated CAG repeat length-dependent alterations in neurodevelopment [47]. However, the absence of MSNs restricted these observations to primarily cortical neuron subtypes [47]. The development of striatal organoids and organoids spanning multiple brain regions could allow for the study of the complex interactions that occur between MSNs and other neuronal and non-neuronal types and provide further insight into non-cell-autonomous mechanisms underlying HD. For example, the generation of human forebrain organoids could shed light on the BDNF deficit hypothesis of HD in a live, human, cellular model of cortico-striatal tracts [117,119,188]. Recently, Miura et al. generated and assembled human striatal and cortical organoids from iPSCs to form cortico-striatal assembloids containing functionally mature MSNs and synaptic connections between cortical neurons and MSNs [189]. Significantly, they observed that cortico-striatal assembloids generated from individuals with a neurodevelopmental disorder demonstrated calcium activity deficits, highlighting the feasibility of this approach to investigate neurodevelopmental and neuronal maturation phenotypes along the cortico-striatal track in patient-derived reprogrammed cells. 

However, the use of mixed cell culture systems to model HD would need to coincide with the utilisation of technologies capable of assessing whether these models are capable of recapitulating human neurodevelopment and HD neuropathogenesis that occurs in vivo, such as single-cell RNA sequencing. 

### 4.7. The Combination of Cell Reprogramming and Animal Models of Huntington’s Disease to Model Pathologies that May Be Dependent on the Specific In Vivo Environment

It is essential to recognise the limitations of in vitro culture systems in recapitulating HD phenotypes that may be dependent on the in vivo microenvironment and complex cell-to-cell interactions that cannot be entirely represented in vitro. While the ability of direct reprogramming to give rise to HD neurons containing the hallmark pathological aggregates present in HD human post-mortem brain tissue provides support for the maintenance of accumulated and later-stage HD pathologies in these models, screening for therapeutic inventions against aggregate formation in vitro may have limited translatability to in vivo systems. In vitro protein folding relies on the intrinsic properties of the protein itself to guide the correct folding of the protein [190], whereas in vivo protein folding is influenced by a range of biological factors, including temporal and spatial limitations in the cells themselves and the correct activity of molecular chaperones [191]. This may explain why very few in vitro models of HD demonstrate transcriptional dysregulation that is dependent on the interactions between abnormally processed mHTT and transcriptional regulators [192,193,194,195]. Targeting abnormal protein folding using cell reprogramming models of HD may benefit from the use of slice culture systems or in vivo transplantation studies, such as those used by Jeon et al. which resulted in the formation of aggregates in HD iPSC-derived neurons after the transplantation of HD NPCs into QA-lesioned rodents [56]. This system may also shed light on the in vivo conditions that contribute to mHTT-associated transcriptional dysregulation.

## 5. Conclusions

Ground-breaking advances in the field of cell reprogramming have substantially benefitted the HD field, providing access to live human HD-affected MSN-like neurons that would otherwise be unobtainable from individuals with HD. Nevertheless, the enormous potential of cell reprogramming depends upon on how effective cell reprogramming models are in representing the in vivo neuropathogenesis of human neurological conditions in vitro. Cell reprogramming models of HD must be interpreted with respect to the methods used to generate the model, the capacity of the model to represent disease neuropathogenesis as it occurs in vivo, and the suitability and appropriateness of the model for downstream applications. The recent ability to generate neurons using direct cell reprogramming represents a powerful tool for studying HD in a system that bypasses epigenetic reset and the return to an hESC-like state that occurs in traditional iPSC reprogramming. Ultimately, the development of a well-characterised cell reprogramming model of HD may prove essential in the ability to study HD in a physiologically relevant system and could provide a novel platform upon which therapeutic interventions for HD could be screened for and tested.

## Figures and Tables

**Table 1:** Studies that have generated neurons from Huntington’s disease human embryonic stem cells.

Study	HD Line	Differentiation	Huntington’s Disease Phenotype
Niclis et al. (2009) [6]	37 CAG 51 CAG	Subpopulation TUJ1+, MAP2+, GFAP+	CAG repeat length instability upon differentiation No difference in neuronal yield
Bradley et al. (2011) [8]	40 CAG 45 CAG 46 CAG 48 CAG	Subpopulation MAP2+	No HD phenotype reported
Feyeux et al. (2012) [20]	40 CAG 44 CAG 44 CAG 46 CAG 48 CAG 51 CAG	Subpopulation MAP2+	No difference in neural rosette formation and expansion Reduced *HTT* expression in NPCs and not neurons Altered expression of mitochondrial function genes and proteins in NPCs
Niclis et al. (2013) [18]	37 CAG 51 CAG	Subpopulation GABA+, TUJ1+	Increased glutamate-evoked responses and increased intracellular calcium levels in neurons (51 CAG) No difference in HD-associated gene expression in hESCs, NPCs, neurons (*HTT*, *BDNF*, *DRD2*, *PPARGC1A*) No difference in neural growth
Lu et al.(2013) [17]	73 CAG * 145 CAG *	Subpopulation TUJ1+	EM48+ HTT aggregates in neurons Increased neuronal cell death upon growth factor deprivation
McQuade at al. (2014) [19]	40 CAG 41 CAG 42 CAG 45 CAG 46 CAG 46 CAG 48 CAG	50% MAP2+ <30% GAD65/67+ (normal CAG) >40% GAD65/67+ (HD CAG)	Significantly more GAD65/67+ cells when differentiated Significantly increased end and branched nodes with no change in neurite length in neurons Increased vulnerability to kinase inhibition in neurons Cytoskeletal and chromatin abnormalities in neurons hESC mitochondrial dysfunction
Ruzo et al. (2018) [16]	45 CAG * 50 CAG * 58 CAG * 67 CAG * 74 CAG *	Subpopulation MAP2+, Nestin+	Impaired formation of neural rosettes during neural induction Dysregulation of mitotic spindle orientation in hESCs Enlarged cell somas and multiple nuclei in progenitors and neurons Severe aneuploidy correlated with CAG repeats Aggregation of cytoplasmic and nuclear HTT in neurons
Cohen-Carmon et al. (2020) [22]	41 CAG 45 CAG 46 CAG 48 CAG	40% GABAergic	Differential expression of genes involved in synapse assembly, glutamatergic synaptic transmission and receptor signalling, and axonogenesis in HD neurons treated with progerin Differential expression of IGF1 in HD only with progerin Upregulation of GAD1 in HD following progerin

* Genetically modified normal hESCs to generate hESCs with HD CAG repeat lengths.

**Table 2:** Studies that have utilized human Huntington’s disease induced pluripotent stem cells.

Adult HD	Age of Onset	Method	Reprogramming Factors	Studies
44/42 CAG	≤59 years	Lentivirus	*OCT4, SOX2, KLF4, cMYC*	Camnasio et al. (2012) [24]
43/39 CAG	≤44 years	Lentivirus	*OCT4, SOX2, KLF4, cMYC*	Camnasio et al. (2012) [24]
40 CAG	Not reported	Lentiviral	*OCT4, SOX2, KLF4, cMYC*	Nekrasov et al. (2016) [25] Nekrasov et al. (2018) [26] Vigont et al. (2018) [27] Vigont et al. (2021) [28]
42 CAG	Not reported	Lentiviral	*OCT4, SOX2, KLF4, cMYC*	Nekrasov et al. (2016) [25] Nekrasov et al. (2018) [26] Vigont et al. (2018) [27] Vigont et al. (2021) [28]
		Sendai virus	*OCT4, SOX2, KLF4, cMYC*	Mollica et al. (2018) [29]
43 CAG	Not reported	Episomal	*OCT4, SOX2, KLF4, cMYC, NANOG*	Chiu et al. (2015) [30]
44 CAG	Not reported	Sendai virus	*OCT4, SOX2, KLF4, cMYC*	Mollica et al. (2018) [29]
			*OCT4, SOX2, KLF4, LMYC, LIN28,* p53 shRNA	Machiela et al. (2020) [31]
45 CAG	≤36 years	Lentivirus	*OCT4, SOX2, KLF4*	Camnasio et al. (2012) [24]
46 CAG	Adult-onset	Episomal	*OCT4, SOX2, KLF4, LMYC, LIN28,* p53 shRNA	Liu et al. (2017) [32] Smith-Geater et al. (2020) [33]
47 CAG	Not reported	Episomal	*OCT4, SOX2, KLF4, LMYC, LIN28, Trp53*	Malankhanova et al. (2020) [34]
		Lentiviral	*OCT4, SOX2, KLF4, cMYC*	Nekrasov et al. (2016) [25] Nekrasov et al. (2018) [26] Vigont et al. (2018) [27] Vigont et al. (2021) [28]
		Retroviral	*OCT4, SOX2, KLF4, cMYC*	Yao et al. (2015) [35]
50 CAG	Not reported	Episomal	*OCT4, SOX2, KLF4, LMYC, LIN28,* p53 shRNA	The HD iPSC Consortium (2019) [36]
53 CAG	Adult-onset	Episomal	*OCT4, SOX2, KLF4, LMYC, LIN28,* p53 shRNA	Grima et al. (2017) [37] Smith-Geater et al. (2020) [33]
57 CAG	≤19 years	Episomal	*OCT4, SOX2, KLF4, LMYC*, p53 shRNA	Tidball et al. (2016) [38]
	26 years		*OCT4, SOX2, KLF4, LMYC*, *LIN28*	Koyuncu et al. (2018) [39]
58 CAG	≤20 years	Episomal	*OCT4, SOX2, KLF4, LMYC,* p53 shRNA	Tidball et al. (2016) [38] Joshi et al. (2019) [40]
**Juvenile HD**	**Age of Onset**	**Method**	**Reprogramming Factors**	**Studies**
60 CAG	18 years	Episomal	*OCT4, SOX2, KLF4, LMYC, LIN28,* p53 shRNA	Mattis et al. (2015) [41] Dickey et al. (2016) [42] The HD iPSC Consortium (2017) [43] Mathkar et al. (2019) [44] The HD iPSC Consortium (2019) [36]
		Lentiviral	*OCT4, SOX2, KLF4, LMYC, LIN28, NANOG*	The HD iPSC Consortium (2012) [45] Martin-Flores et al. (2015) [46] Conforti et al. (2018) [47]
66 CAG	≤20 years	Episomal	*OCT4, SOX2, KLF4, LMYC, LIN28,* p53 shRNA	The HD iPSC Consortium (2019) [36] Smith-Geater et al. (2020) [33] Morozko et al. (2021) [48]
69 CAG	Not reported	Episomal	*OCT4, SOX2, KLF4, LMYC, LIN28*, p53 shRNA	Liu et al. (2017) [32]
70 CAG	≤50 years	Retroviral	*OCT4, SOX2, KLF4, cMYC*	Yao et al. (2015) [35] Cohen-Carmon et al. (2020) [22]
	Not reported	Episomal	*OCT4, SOX2, KLF4, LMYC*	Tidball et al. (2015) [49]
			*OCT4, SOX2, KLF4, LMYC*, p53 shRNA	Tidball et al. (2016) [38] Joshi et al. (2019) [40]
			*OCT4, SOX2, KLF4, LMYC, LIN28,* p53 shRNA	Liu et al. (2017) [32]
71 CAG	14 years	Episomal	*OCT4, SOX2, KLF4, LMYC, LIN28*, p53 shRNA	Mattis et al. (2015) [41] Szlachcic et al. (2015) [50] Koyuncu et al. (2018) [39] Switonska et al. (2019) [51] Mathkar et al. (2019) [44] Smith-Geater et al. (2020) [33] Machiela et al. (2020) [31] Morozko et al. (2021) [48]
72 CAG	14 years	Retroviral	*OCT4, SOX2, KLF4, cMYC*	Park et al. (2008) [52] Zhang et al. (2010) [53] An et al. (2012) [54] Chae et al. (2012) [55] Jeon et al. (2012) [56] Charbord et al. (2013) [57] Cheng et al. (2013) [58] Ring et al. (2015) [59] Naphade et al. (2018) [60] Lopes et al. (2020) [61] Le Cann et al. (2021) [62]
76 CAG	≤17 years	Sendai virus	*OCT4, SOX2, KLF4, LMYC*	Vigont et al. (2021) [28]
		Lentiviral	*OCT4, SOX2, KLF4, LMYC*	Vigont et al. (2021) [28]
77 CAG	Juvenile	Episomal	*OCT4, SOX2, KLF4, LMYC, LIN28*, p53 shRNA	Mehta et al. (2018) [63] Mathkar et al. (2019) [44]
99 CAG	Not reported	Episomal	*OCT4, SOX2, KLF4, LMYC, LIN28*, p53 shRNA	Liu et al. (2017) [32]
100 CAG *	2 years	Lentiviral	*OCT4, SOX2, KLF4, cMYC*	Guo et al. (2013) [64]
109 CAG	3 years	Episomal	*OCT4, SOX2, KLF4, LMYC, LIN28,* p53 shRNA	Lu et al. (2014) [65] Mattis et al. (2015) [41] Szlachcic et al. (2015) [50] Grima et al. (2017) [37] The HD iPSC Consortium (2017) [43] Mehta et al. (2018) [63] Switonska et al. (2019) [51] Mathkar et al. (2019) [44] The HD iPSC Consortium (2019) [36] Smith-Geater et al. (2020) [33] Machiela et al. (2020) [31] Morozko et al. (2021) [48] Le Cann et al. (2021) [62]
		Lentiviral	*OCT4, SOX2, KLF4, LMYC, LIN28, NANOG*	The HD iPSC Consortium (2012) [45] Conforti et al. (2018) [47] Aron et al. (2018) [66]
180 CAG	6 years	Retroviral	*OCT4, SOX2, KLF4, cMYC*	Cohen-Carmon et al. (2020) [22]
		Lentiviral	*OCT4, SOX2, KLF4, LMYC, LIN28, NANOG*	The HD iPSC Consortium (2012) [45] Ooi et al. (2015) [67] Xu et al. (2017) [68] Conforti et al. (2018) [47]
		Episomal	*OCT4, SOX2, KLF4, LMYC*	Tidball et al. (2015) [49]
			*OCT4, SOX2, KLF4, LMYC, LIN28*, p53 shRNA	Lu et al. (2014) [65] Mattis et al. (2015) [41] Mehta et al. (2017) [63] Mathkar et al. (2019) [44] Smith-Geater et al. (2020) [33] Le Cann et al. (2021) [62]
			*OCT4, SOX2, KLF4, LMYC*, p53 shRNA	Tidball et al. (2016) [38] Koyuncu et al. (2018) [39]

* CAG repeat length not reported; genotyped to approximately 100 CAG repeats by Zhao et al. [69] and Evers et al. [70].

**Table 3:** Phenotypes observed in Huntington’s disease induced pluripotent stem cells.

Study	HD Line	Huntington’s Disease Phenotype
Zhang et al. (2010) [53]	72 CAG	Decreased ERK phosphorylation in response to FGF
An et al. (2012) [54]	72 CAG	Increased expression of caspase signalling genes Increased expression of TGFβ signalling genes Altered expression of cadherin family genes No difference in proliferation rate No difference in caspase-3/7 after growth factor removal
Chae et al. (2012) [55]	72 CAG	Increased expression of oxidative stress genes Decreased expression of oxidative stress response proteins Increased expression of antioxidant response proteins Protein changes in factors regulating p53, NFκB
Camnasio et al. (2012) [24]	44/42 CAG 43/39 CAG 45 CAG	No difference in the number of mitotic cells Increased lysosomal activity in response to sucrose No difference in caspase-3/7 expression
Tidball et al. (2015) [49]	70 CAG 180 CAG	Increased expression of p53, phosphorylated p53, phosphorylated ATM
Szlachcic et al. (2015) [50]	71 CAG 109 CAG	Increased expression of oxidative stress factor SOD1 Decreased ERK phosphorylation (no change β-catenin)
Tidball et al. (2016) [38]	57 CAG 58 CAG 70 CAG 180 CAG	Increased chromosomal abnormalities after reprogramming (stable with continuous passaging) Neocarzinostatin protected against induced DNA damage
Liu et al. (2017) [32]	46 CAG 69 CAG 70 CAG 99 CAG	Increased proteolytic and proteasome activity Increased expression of *FOXO4* senescence gene
Mollica et al. (2018) [29]	42 CAG 44 CAG	CAG repeat length stable throughout reprogramming Reprogramming corrected DNA repair gene expression
Switonska et al. (2018) [51]	71 CAG 109 CAG	Altered expression of embryonic development genes Decreased expression of DNA damage response genes Dysregulated expression of p53 protein
The HD iPSC Consortium (2019) [36]	50 CAG 60 CAG 66 CAG 109 CAG	Decreased ATP levels in CAG-dependent manner
Joshi et al. (2019) [40]	58 CAG 70 CAG	HD more resistant to Mn cytotoxicity
Lopes et al. (2020) [61]	72 CAG	Altered mitochondrial morphology Reduced number of mitochondria Reduced mitochondrial area Reduced OPA1 co-localization with mitochondria Exosomes enriched with ATP synthesis, apoptosis signalling, and synaptic vesicle trafficking proteins Significantly decreased basal respiration rate Increased dependence on glycolysis Increased basal intracellular calcium levels Reduced *PGC1α* and *TFAM* mRNA expression Increased basal superoxide levels Increased GSH expression and *GCLc* mRNA

**Table 4:** Phenotypes observed in Huntington’s disease neural precursor cells derived from induced pluripotent stem cells.

Study	HD Line	Markers	Huntington’s Disease Phenotype
Zhang et al. (2010) [53]	72 CAG	Nestin, SOX1, PAX6	Growth factor withdrawal (EGF) increased caspase-3/7
An et al. (2012) [54]	72 CAG	Nestin	Decreased expression of *TGFβ* and *BDNF* Decreased expression of n-cadherin gene and protein Decreased mitochondrial respiration rate after uncoupling treatment Growth factor withdrawal (FGF/LIF) increased cell death and caspase-3/7 Phenotypes reversed in isogenic corrected cells
The HD iPSC Consortium (2012) [45]	60 CAG 109 CAG 180 CAG	Nestin, PAX6, SOX1, SOX2	Altered expression of cell signalling, cell cycle, cell assembly, embryonic development, cell development genes Altered expression of axon guidance genes (109 CAG, 180 CAG) Altered expression of calcium signalling genes (60 CAG) Decreased expression of actin protein Decreased intracellular ATP and decreased ATP/ADP ratio Decreased sphere formation after single-cell suspension Increased cleaved caspase-3 (180 CAG)
Camnasio et al. (2012) [24]	44/42 CAG 43/39 CAG 45 CAG	Not reported	Increased lysosomal activity
Jeon et al. (2012) [56]	72 CAG	Nestin	Form EM48+ aggregates 40 weeks after transplantation into QA rats
Charbord et al. (2013) [57]	72 CAG	Not reported	Decreased expression of *BDNF* REST inhibitor X5050 reduced REST inhibitor, increased *RE1* expression, *SNAP25*, *BDNF*, *SYP* gene expression and activity normalized to ESCs
Tidball et al. (2015) [49]	70 CAG 180 CAG	ISLET, PAX6, FOXG1	Mn^2+^ treatment decreased phosphorylated p53 and phosphorylated AKT Decreased Mn^2+^ uptake following Mn^2+^ treatment No change in caspase-3 activation following Mn^2+^ treatment Induced DNA breakage increased phosphorylated p53 and H2AX ATM inhibition reversed DNA breakage and Mn^2+^ induced phenotypes
Ring et al. (2015) [59]	72 CAG	Nestin	Altered expression of axon guidance and synapse assembly genes Altered expression of genes regulating TGFβ expression Decreased expression of striatal genes (*DARPP32*, *CTIP2*, *FOXP1, ISL1)* Decreased expression of dorsal forebrain genes (*TBR1*, *PAX6)* Increased expression of striatal gene *FOXP2* and *NETRIN* Altered expression of netrin receptor genes Growth factor withdrawal (FGF/LIF) increased caspase-3/7 activation and decreased maximal respiratory capacity reversed by TGFβ netrin
Ooi et al. (2015) [67]	180 CAG	Nestin	Decreased expression of *MAO-A* and *MAO-B* Increased activity of MAO-A and MAO-B
Martin-Flores et al. (2016) [46]	60 CAG	Not reported	Increased expression of AKT/mTOR signalling effector protein
Xu et al. (2017) [68]	180 CAG	Nestin	Impaired formation of neural rosettes
Liu et al. (2017) [32]	46 CAG 69 CAG 70 CAG 99 CAG	Nestin, SOX2	Reduced proteasome activity Reduced expression of *FOXO4* senescence gene Increased expression of ubiquitin genes
Naphade et al. (2017) [60]	72 CAG	Nestin	Altered expression of metalloproteinases genes Increased expression of TGFβ protein Exogenous TGFβ reversed mitochondrial defects and reduced caspase-3/7
Mollica et al. (2018) [29]	42 CAG 44 CAG	Nestin, SOX2, SOX1, PAX6	CAG repeat length stable throughout reprogramming
Conforti et al. (2018) [47]	60 CAG 109 CAG 180 CAG	Nestin, SOX2	Inability to acquire a neuroectodermal identity (180 CAG) Abnormalities in neural rosette formation
The HD iPSC Consortium (2019) [36]	50 CAG 60 CAG 66 CAG 109 CAG	Not reported	Decreased ATP levels in CAG-dependent manner Increased susceptibility to oxidative stress caused by hydrogen peroxide
Joshi et al. (2019) [40]	58 CAG 70 CAG	PAX6, SOX1 or ISLET1	Increased protection against Mn cytotoxicity in striatal and cortical NPCs in HD
Lopes et al. (2020) [61]	72 CAG	Nestin, SOX2	Altered mitochondrial morphology Reduced number of mitochondria Reduced mitochondrial area Reduced OPA1 co-localization with mitochondria Exosomes enriched with ATP synthesis and TCA cycle proteins Significantly decreased basal respiration rate Significantly decreased maximal and spare respiratory capacity Increased dependence on glycolysis Decreased basal intracellular calcium levels Reduced *PGC1α* mRNA expression Increased basal superoxide levels Increased SOD2 activity upon neural induction Increased *NRF2* mRNA upon neural induction and compared to control Basal respiration and mitochondrial ROS levels rescued by genetic correction
Malankhanova et al. (2020) [34]	47 CAG 69 CAG *	PAX6, SOX1, OTX2	Impaired formation of neural rosettes

* Normal human embryonic fibroblasts genetically modified to induce a 69 CAG repeat expansion in the HD gene and were subsequently reprogrammed into iPSCs.

**Table 5:** Phenotypes observed in Huntington’s disease neurons derived from induced pluripotent stem cells.

Study	HD Line	Differentiation	Huntington’s Disease Phenotype
The HD iPSC Consortium (2012) [45]	60 CAG 109 CAG 180 CAG	<10% TUJ1+, <10% MAP2+, <5% DARPP32+/TUJ1+	Altered expression of genes involved in cell growth, proliferation, cell function, cell-to-cell signalling, and embryonic development Impaired electrophysiological activity (180 CAG) Increased cell death (condensed nuclei) (180 CAG) Increased risk of cell death (60 CAG, 180 CAG) reversed by 4x BDNF concentration Increased cell death (increased condensed nuclei) (180 CAG) with autophagy and not proteasome inhibition Increased calcium dyshomeostasis with glutamate pulse (60 CAG) Increased cell death following glutamate pulse (60 CAG, 180 CAG) Increased cell death upon BDNF withdrawal (109 CAG, 180 CAG) Increased caspase-3/7 activity with BDNF withdrawal CAG repeat length instability (110 CAG to 118 CAG)
Chae et al. (2012) [55]	72 CAG	36% MAP2+ (HD) 51% MAP2+ (normal)	Decreased expression of cytoskeletal proteins Decreased expression of oxidative stress response proteins Increased expression of anti-oxidant response proteins Increased expression of double-strand DNA damage response genes Increased expression and phosphorylation of ATM, p53, H2AX Increased cleavage of apoptosis proteins (caspase-3, caspase-7) Reduced yield and neurite length of MAP2+ neurons Increased cell death
Camnasio et al. (2012) [24]	44/42 CAG 43/39 CAG 45 CAG	12–34% TUJ1+, subpopulation GABA+, GAD65/67+, MAP2+	Increased macroautophagy
Cheng et al. (2013) [58]	72 CAG	Subpopulation TUJ1+	EM48+ aggregates induced by proteasome inhibition reduced by lentiviral miR-196
Guo et al. (2013) [64]	100 CAG	Subpopulation DARPP32+, GAD67+, TUJ1+, MAP2+	Decreased mitochondrial membrane potential and ATP Increased mitochondrial ROS production Increased cell death (lactate dehydrogenase increased) Increased mitochondrial fragmentation in GAD67+ neurites Shorter neurites in GAD67+ and DARPP32+ neurons DRP1 inhibitor reversed mitochondrial fragmentation, increased neurite length in GAD67+ and DARPP32+ neurons, increased mitochondrial membrane potential, decreased mitochondrial ROS, increased ATP, decreased cell death p53 shRNA increased neurite length in GAD67+ and DARPP32+ neurons, increased mitochondrial membrane potential, decreased mitochondrial ROS, decreased cell death
Lu et al. (2014) [65]	109 CAG 180 CAG	5% DARPP32+, 10% TUJ1+	Increased cell death upon BDNF withdrawal Phenotype reversed by ATM inhibition
Chiu et al. (2015) [30]	43 CAG	70% GABA+, GAD65+, DARPP32+, Calbindin+ of TUJ1+, MAP2+	Decreased expression of DNA damage response, G-protein signalling, oxidative stress repair genes Increased expression of striatal genes (DRD1/2, GAD65) Reduced adenosine receptor gene and protein expression No difference in caspase-3 activity No difference in levels of phosphorylated H2AX Increased expression of H2AX after hydrogen peroxide Phenotype reversed by adenosine receptor antagonism or protein kinase A (PKA) inhibition
Yao et al. (2015) [35]	47 CAG 70 CAG	Subpopulation TUJ1+, DARPP32+, GABA+	G-protein signalling receptor knockdown reduced mHTT BDNF withdrawal-induced caspase-3 activation and neuronal loss reduced by G-protein signalling receptor knockdown G-protein signalling receptor knockdown increased neurite complexity
Mattis et al. (2015) [41]	60 CAG 109 CAG 180 CAG	5% TUJ1+ (normal) 4% DARPP32+ (normal) 20% TUJ1+ (HD) 1–4% DARPP32+ (HD)	Increased expression of GRIN2B NMDA receptor subunit mRNA (180 CAG and 109 CAG, no change in 60 CAG) with BDNF withdrawal Increased persistence of Nestin+ cells when differentiated Nestin+ cells more susceptible to cell death on BDNF withdrawal BDNF withdrawal-induced increased cell death BDNF withdrawal-induced cell death reversed by calcium chelator, TrkB receptor agonist, MAPK signalling inhibitor, NMDA receptor antagonism, AMPA receptor antagonism (109 CAG, 180 CAG)
Nekrasov et al. (2016) [25]	40 CAG 42 CAG 47 CAG	93% TUJ1+, 79% DARPP32+/TUJ1+	Proteasome inhibition increased mHTT aggregation Increased number of autophagosomes Increased lysosomal activity and number of lysosomes SOC-mediated calcium dysfunction Phenotype reversed by NF-κB inhibition
Dickey et al. (2017) [42]	60 CAG	Subpopulation TUJ1+, DARPP32+	PPARδ activator reversed BDNF withdrawal-induced cell death
Grima et al. (2017) [37]	53 CAG 109 CAG	Subpopulation MAP2+	Localization of nucleoporins and nuclear pore complexes to cytoplasm
The HD iPSC Consortium (2017) [43]	60 CAG 109 CAG	27% TUJ1+, 15% MAP2+, 14% DARPP32+	Decreased expression of calcium signalling pathway genes (NMDA receptor subunit GRIN2B, AMPA receptor, CREB, CACNA1C, CAMK) Decreased expression of striatal development genes (ASCL1, GAD1, GAD2) and neurodevelopment genes (NEUROG1, NEUROG2, HES5) Increased expression of TGFβ receptor family genes Epigenetic and chromatin alterations Reversed by neurogenesis enhancer isoxazole-9
Xu et al. (2017) [68]	180 CAG	Subpopulation GABA+, GAD65+, MAP2+	Increased susceptibility to growth factor withdrawal and mitochondrial dysfunction corrected by isogenic controls
Liu et al. (2017) [32]	46 CAG 69 CAG 70 CAG 99 CAG	Subpopulation DARPP32+, GABA+, TUJ1+	Increased cell death following oxidative stress Reduced proteasome activity Reduced expression of FOXO4 senescence gene Reduced expression of phosphorylated AKT
Conforti et al. (2018) [47]	60 CAG 109 CAG 180 CAG	Subpopulation DARPP32+, CTIP2+, TUJ1+, MAP2+	Impaired acquisition of striatal progenitor identity and maturation Decreased neuronal yield correlated with CAG repeat length Reduced expression of striatal genes *(GSX2, DARPP32/PPP1R1B)* Altered expression of electrophysiological genes Phenotype reversed metalloproteases inhibitor
Mehta et al. (2018) [63]	77 CAG 109 CAG 180 CAG	25–30% MAP2+, TUJ1+ 20% CTIP2+, TBR1+ of MAP2+, TUJ1+	Altered expression of neuronal maturation and morphology genes Reduced voltage-gated sodium current gene expression Reduced neurite length correlated with CAG repeat length Immature RMPs (−30 mV) compared to normal (−45 mV) with no improvement over time (normal decreased over time)
Nekrasov et al. (2018) [26]	40 CAG 42 CAG 47 CAG	Subpopulation TUJ1+, striatal differentiation protocol	Reduced neurite mitochondrial density Proteasome inhibition exacerbated reduced neurite mitochondrial density Nuclear calcium homeostasis disruption did not alter HD pathology
Vigont et al. (2018) [27]	40 CAG 42 CAG 47 CAG	Subpopulation GABA+	Upregulation of store-operated calcium entry EVP4593 had a neuroprotective effect on HD
The HD iPSC Consortium (2019) [36]	50 CAG 60 CAG 66 CAG 109 CAG	90% MAP2+, 20–30% DARPP32+/MAP2+, 60–80% CTIP2+/MAP2+	Decreased ATP levels in CAG-dependent manner Decreased expression of glycolysis genes and proteins Increased expression of oxidative phosphorylation proteins (not mRNA) Deficit in maximal mitochondrial respiration (109 CAG) Decreased glycolytic activity with ATP synthase inhibitor Increased ATP production with pyruvate treatment
Mathkar et al. (2019) [44]	60 CAG 71 CAG 77 CAG 109 CAG 180 CAG	10–20% TUJ1+, MAP2+	Persistent Nestin+ cells in differentiated cell population (juvenile) Reduced GFAP+ cells in late differentiation (juvenile) No difference in proliferation of differentiated cells (juvenile) Phenotype reversed by DAPT or HTT knockdown
Joshi et al. (2019) [40]	58 CAG 70 CAG	Subpopulation TUJ1+, Glutamate+, GFAP+, TH+	No difference in Mn sensitivity in cortical HD neurons Increased Mn sensitivity in midbrain HD neurons
Cohen-Carmon et al. (2020) [22]	70 CAG 180 CAG	40% GABAergic	Differential expression of genes involved in cilium organization and neurogenesis in HD following progerin treatment Differential expression of IGF1 in HD only with progerin treatment Upregulation of GAD1 in HD with progerin treatment
Smith-Geater et al. (2020) [33]	46 CAG 53 CAG 66 CAG 71 CAG 109 CAG 180 CAG	79–98% MAP2+, 4–28% DARPP32+	Persistent cyclin D1+ NSC-like population in adult HD Wnt inhibition abrogates persistent NSC-like populations Upregulation of cell cycle related genes in HD
Malankhanova et al. (2020) [34]	47 CAG 69 CAG *	Subpopulation of DARPP32+, GABA+, TUJ1+	BDNF-withdrawal induced cell death and caspase-3 Dendritic spine density and length morphology- dependent Increased presence of cytoplasmic vacuoles Aggregates of autophagosomes and autolysosomes
Machiela et al. (2020) [31]	43 CAG 71 CAG 109 CAG	Subpopulation TUJ1+, striatal differentiation protocol	AAV-transduced progerin in NSCs results in reduced dendritic length in differentiated cells from both control and HD AAV-transduced progerin in NSCs results in increased DNA damage and γH2AX foci in differentiated HD but not differentiated control cells
Le Cann et al. (2021) [62]	72 CAG 109 CAG 180 CAG	0–64% DARPP32+/GAD+ 0–40% GAD67+, 9–40% TUJ1+	Navβ4 subunit expression unreliable HD biomarker Action potential formation unaffected by HD genotype Greater differences observed between two alternate striatal differentiation protocols than between HD and control lines within either protocol
Morozko et al. (2021) [48]	66 CAG 71 CAG 109 CAG	Subpopulation DARPP32+, CTIP2+, MAP2+	Enriched synaptic signalling with PIAS1 knockdown in control and HD PIAS1 knockdown increased genomic integrity and DNA damage repair
Vigont et al. (2021) [28]	40 CAG 42 CAG 47 CAG 76 CAG	80% DARPP32, ≤100% MAP2+	Dramatically disrupted calcium signalling (76 CAG) Calcium signalling alterations do not depend on CAG repeat length Increased STIM2 expression mediating SOC entry into neurons (76 CAG) HTT and STIM2 expression attenuated by EVP4593 (76 CAG)

* Normal human embryonic fibroblasts genetically modified to induce a 69 CAG repeat expansion in the HD gene and were subsequently reprogrammed into iPSCs.

**Table 6:** Phenotypes observed in Huntington’s disease directly induced neurons.

Study	HD Line	Differentiation	Huntington’s Disease Phenotype
Liu et al. (2014) [111]	68 CAG (onset 14 years) 86 CAG * (onset 2 years)	8% TUJ1+ (normal) <40% DARPP32+ (normal) 8% TUJ1+ (HD) 60% DARPP32+ (HD)	Abnormal neurite branching and neurite degeneration Increased loss of GABA+ cells Increased apoptotic cell death Nuclear and cytoplasmic HTT aggregates
Drouin- Ouellet et al. (2017) [109]	41 CAG (onset ≤ 58 years)	25–62% MAP2+	Impaired neuronal conversion (corrected by removal of cell passaging) No difference in neurite number or ratio of bipolar/multipolar neurons No difference in expression of synaptic genes or neuronal genes
Victor et al. (2018) [110]	40 CAG (onset 63 years) 42 CAG (onset ≤ 51 years) 43 CAG (onset 50 years) 44 CAG (onset ≤ 52 years) 46 CAG (onset ≤ 60 years) 47 CAG (onset ≤ 63 years) 50 CAG (onset 27 years)	90% MAP2+, 70–80% GABA+, 70–80% DARPP32+	CAG repeat length stable throughout the time course Increased multiple action potentials (rat glia co-culture) Altered expression of differentiation, calcium signalling, apoptosis genes Decreased expression of *HAP1, NTRK2,* and *SP9* MSN survival gene Increased expression of synaptic genes (*KCNA4*, GABA/AMPA receptors) Nuclear and cytoplasmic HTT aggregates (including pre-symptomatic HD) No aggregates in iPSC-derived MSNs from HD lines Mitochondrial dysfunction, mitophagy Increased rate of neuronal cell death with time in culture Increased oxidative DNA damage Incomplete lysosomal degradation of damaged/dysfunctional mitochondria Increased ROS expression Decreased expression of ubiquitin- proteasome system genes Oxidative stress-induced cell death reversed by ATM inhibition

* CAG repeat length stated by authors as 86 CAG; genotyping conducted by Zhao et al. [69] and Evers et al. [70] determined the approximate CAG repeat length of the original fibroblast line as approximately 100 CAG repeats.

**Table 7:** Phenotypes observed in Huntington’s disease induced neural precursor cell-derived neurons.

Study	HD Line	Differentiation	Huntington’s Disease Phenotype
Hou et al. (2017) [133]	41 CAG (onset not reported) 41 CAG (onset not reported)	Subpopulation TUJ1+, GFAP+, GALC+	Increased γH2AX indicating DNA damage in embryonic-like progenitors Adenosine receptor agonist reversed phenotype
Monk et al. (2021) [134]	41 CAG (onset 56 years) 43 CAG (onset 50 years) 44 CAG (onset 33 years) 57 CAG (onset 27 years)	20% TUJ1+ (normal) 12% TUJ1+ (HD) 69% DARPP32+/TUJ1+ (normal) 54% DARPP32+/TUJ1+ (HD)	Ubiquitinated mHTT aggregates (57 CAG) Maintenance of transcriptional phenotype Significantly smaller cell soma areas Significantly shorter neurite length Significantly reduced neurite branching Significantly more depolarized RMPs Decreased BDNF protein correlated with reduced age of symptom onset and increased number of CAG repeats
